# Effects of Diabetes Mellitus on Fibrin Clot Structure and Mechanics in a Model of Acute Neutrophil Extracellular Traps (NETs) Formation

**DOI:** 10.3390/ijms21197107

**Published:** 2020-09-26

**Authors:** Judith J. de Vries, Tamara Hoppenbrouwers, Cristina Martinez-Torres, Rezin Majied, Behiye Özcan, Mandy van Hoek, Frank W.G. Leebeek, Dingeman C. Rijken, Gijsje H. Koenderink, Moniek P.M. de Maat

**Affiliations:** 1 Department of Hematology, Erasmus MC, University Medical Center Rotterdam, 3015 GD Rotterdam, The Netherlands; j.j.devries.1@erasmusmc.nl (J.J.d.V.); t.hoppenbrouwers@yahoo.com (T.H.); r.majied@erasmusmc.nl (R.M.); f.leebeek@erasmusmc.nl (F.W.G.L.); d.rijken@erasmusmc.nl (D.C.R.); 2 Food Quality and Design, Wageningen University & Research, 6708 WG Wageningen, The Netherlands; 3 AMOLF, Living Matter Department, 1098 XG Amsterdam, The Netherlands; c.e.martineztorres@tudelft.nl (C.M.-T.); g.h.koenderink@tudelft.nl (G.H.K.); 4 Department of Bionanoscience, Kavli Institute of Nanoscience, Delft University of Technology, 2629 HZ Delft, The Netherlands; 5 Department of Internal Medicine, Erasmus MC, University Medical Center Rotterdam, 3015 GD Rotterdam, The Netherlands; b.ozcan@erasmusmc.nl (B.Ö.); m.vanhoek@erasmusmc.nl (M.v.H.)

**Keywords:** diabetes mellitus, fibrin, fibrinolysis, neutrophil extracellular traps, arterial thrombosis

## Abstract

Subjects with diabetes mellitus (DM) have an increased risk of arterial thrombosis, to which changes in clot structure and mechanics may contribute. Another contributing factor might be an increased formation of neutrophil extracellular traps (NETs) in DM. NETs are mainly formed during the acute phase of disease and form a network within the fibrin matrix, thereby influencing clot properties. Previous research has shown separate effects of NETs and DM on clot properties, therefore our aim was to study how DM affects clot properties in a model resembling an acute phase of disease with NETs formation. Clots were prepared from citrated plasma from subjects with and without DM with the addition of NETs, induced in neutrophils by *S. aureus* bacteria or phorbol myristate acetate (PMA). Structural parameters were measured using scanning electron microscopy, mechanical properties using rheology, and sensitivity to lysis using a fluorescence-based fibrinolysis assay. Plasma clots from subjects with DM had significantly thicker fibers and fewer pores and branch points than clots from subjects without DM. In addition, fibrinolysis was significantly slower, while mechanical properties were similar between both groups. In conclusion, in a model of acute NETs formation, DM plasma shows prothrombotic effects on fibrin clots.

## 1. Introduction

Diabetes mellitus (DM) is an increasingly common disease with a prevalence of almost 500 million people worldwide and DM is directly responsible for 1.6 million deaths per year [1,2]. It is expected that these high numbers will keep increasing over the coming decades if no effective preventive strategies are implemented [1]. An important part of these deaths and the morbidity associated with DM are caused by arterial thrombotic disorders, such as myocardial infarction or stroke [3]. Since subjects with DM have more compact clots with smaller pores, which have a decreased susceptibility to fibrinolysis [4,5], it is hypothesized that these different structural and mechanical clot characteristics contribute to the increased thrombotic risk. It has indeed been shown that denser clots with smaller pore sizes are more resistant to fibrinolysis and are associated with arterial thrombosis [6]. 

The matrix of the thrombus does not only consist of fibrin fibers, but also contains a network formed by neutrophil extracellular traps (NETs) [7]. NETs are also found to influence clot characteristics and fibrinolysis [8,9] and thereby thrombotic risk [10,11]. NETs are secreted during the acute phase of disease by activated neutrophils as part of the innate immune system and resemble web-like structures, composed of DNA, histones and granular proteins, which kill extracellular pathogens [12]. The pro-coagulant properties of NETs derive from their ability to provide a scaffold for thrombus development, activate coagulation factors and platelets, and inactivate coagulation pathway inhibitors [10,13]. The formation of NETs was shown to be enhanced in different patient groups with increased pro-coagulant activity [14,15,16] and also in patients with DM [17,18]. However, in a large population study, we did not observe differences in NETs plasma levels between subjects with and without DM in steady state conditions [19].

Most research regarding the effects of DM on clot properties is performed with purified fibrinogen or plasma. However, NETs also affect clot properties, while plasma levels of NETs in steady state conditions are generally very low [19]. Therefore, to resemble acute phase conditions in disease, we added a fixed amount of activated neutrophils to plasma from subjects with or without DM before clot formation. The aim of our study was to investigate the effect of DM on structural, mechanical and lysis characteristics of plasma clots in a model of acute NETs formation. This is important, since changes in these clot characteristics have important consequences for the susceptibility to treatment.

## 2. Results

Plasma samples from 20 subjects with DM and 26 subjects without DM of the ATTAC study were used in the current study (Table 1). The mean age of the selected study population was 46.4 ± 5.7 years, 35% of subjects was male and 61% of subjects suffered from arterial thrombosis. 

The body mass index of subjects with DM was significantly higher (29.9 [27.0–36.5]) compared to subjects without DM (27.0 [24.2–29.5]) (*p* = 0.03). Subjects with DM more often used statins (85%) compared to subjects without DM (54%), while no difference was found in the percentage of subjects using aspirin (70% of subjects with DM, 50% of subjects without DM). Plasma levels of fibrinogen, glucose and HbA1c were significantly higher in subjects with DM (Table 1). 

### 2.1. Fibrinogen Concentration Was Significantly Associated with Clot Properties

In our study, we observed a significantly higher concentration of fibrinogen in subjects with DM (3.8 ± 0.7 g/L) compared to subjects without DM (3.3 ± 0.6 g/L) (*p* = 0.01). Since fibrinogen concentration is known to affect clot properties [20], we first determined the correlation of the fibrinogen concentration with the clot parameters in a model of acute NETs formation. Fibrinogen levels were positively associated with fiber diameter (Spearman’s correlation coefficient (r_s_) = 0.60, *p* = 0.01) (Table 2). Furthermore, fibrinogen levels were positively associated with stiffness of the clots (G’) (r_s_ = 0.55, *p* < 0.01), onset stress for strain-stiffening (σ_0_) (r_s_ = 0.56, *p* < 0.01) and the maximum elastic modulus, K_max_, reached before clot rupture (r_s_ = 0.56, *p* < 0.01), while a negative association was found between fibrinogen levels and the onset strain for strain-stiffening γ_0_ (r_s_ = −0.35, *p* = 0.03) and the strain at the point of rupture γ_R_ (r_s_ = −0.40, *p* = 0.01). This implicates that higher fibrinogen concentrations resulted in stiffer clots over the entire range of shear strains. Fibrinogen concentration was not significantly associated with the stress and strain levels at K_max_ and stress at rupture (σ_R_). Finally, fibrinogen concentration was negatively associated with the rate of fibrinolysis (r_s_ = −0.52, *p* < 0.01). Because of the association of fibrinogen concentration with both DM and the clot parameters, subsequent analyses are adjusted for fibrinogen levels. 

Glucose and HbA1c levels were not significantly associated with mechanical parameters of the clots, except for a weak correlation of glucose with K_max_ (r_s_ = 0.41, *p* = 0.04) (Table 2). Fiber diameter was positively associated with glucose (r_s_ = 0.74, *p* = 0.04) and HbA1c levels (r_s_ = 0.83, *p* = 0.01), while a negative association was found between HbA1c levels and the number of pores (r_s_ = −0.71, *p* = 0.05) and intersection density (r_s_ = −0.74, *p* = 0.04).

### 2.2. Clot Network Characteristics in Plasma Clots with NETs in Subjects with and without DM

**Figure 1:**
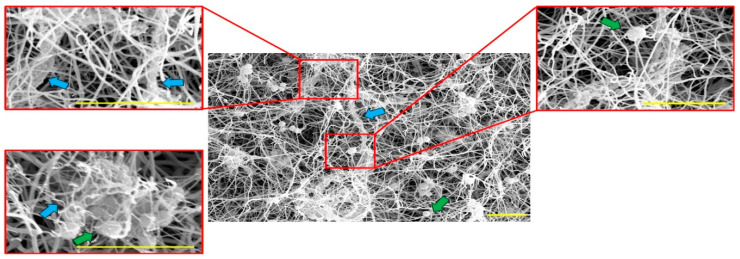
SEM images of clots prepared from plasma with addition of neutrophils, activated by the *S. aureus* bacteria. The bacteria are visible as small circular shapes in the images (green arrows), often entrapped by the fibrin or NETs network. Very thin fibers which form dense networks or tube-like shapes within the fibrin network are visible, which we suspect to be NETs (blue arrows). Scale bars 5 µm.

**Figure 2:**
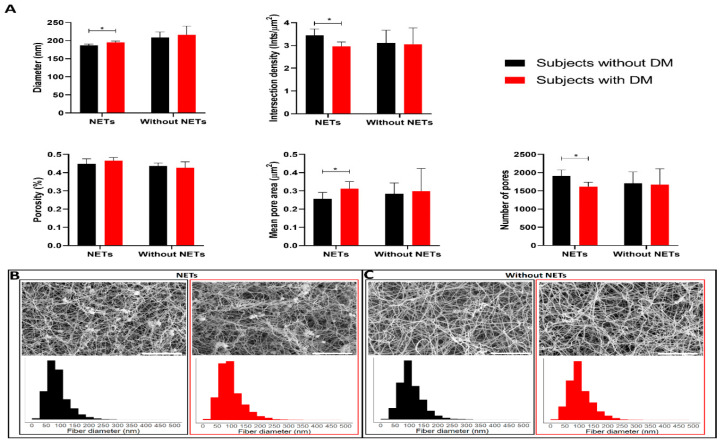
Structural properties of clots prepared from plasma from subjects without or with diabetes mellitus (DM) with or without the addition of neutrophil extracellular traps (NETs) determined in SEM images using the ImageJ plugin DiameterJ. The error bars in (**A**) represent the mean with standard deviation. (**B**,**C**) show representative images and histograms of fiber diameters in plasma clots from a subject without (black) and with DM (red) prepared with or without addition of NETs, respectively. Scale bars 10 µm. * indicates *p*-value below 0.05.

SEM images of plasma clots with added NETs showed very thin fibers that formed dense networks and bundles within the fibrin network, which were not visible in images of clots without added NETs (Figure 1). In clots to which NETs were added, fibers in plasma clots from subjects with DM were significantly thicker (195.0 ± 3.9 nm) compared to fibers in plasma clots from subjects without DM (186.6 ± 3.9 nm, *p* < 0.01) (Figure 2), which was independent of fibrinogen levels (Table 3). 

Furthermore, after adjustment for fibrinogen levels, plasma clots in the presence of NETs from subjects with DM had a decreased number of pores (beta coefficient [B] −268.0, 95% CI −472.6;−63.4) and a lower intersection density (B −0.43, 95% CI −0.78;−0.09] compared to plasma clots from subjects without DM. 

Thicker fibrin fibers were correlated with increased pore size (r_s_ = 0.50, *p* < 0.05), decreased number of pores (r_s_ = −0.69, *p* < 0.01) and lower intersection density (r = −0.74, *p* < 0.01) (Table S1). Furthermore, porosity was positively correlated with pore size (r_s_ = 0.92, *p* < 0.01) and negatively with the number of pores (r_s_ = −0.82, *p* < 0.01) and intersection density (r_s_ = −0.78, *p* < 0.01).

In clots without the addition of NETs, no significant differences between subjects with (209.1 [203.3–214.0 nm]) and without DM (207.5 [199.6–222.4 nm]) (*p* = 0.66) were found in fiber diameter. Also the porosity, size and number of pores and the intersection density were not different in clots without added NETs between subjects with and without DM (Figure 2 and Table S2). 

### 2.3. Mechanical Parameters of Plasma Clots with NETs in Subjects with and without DM

**Figure 3:**
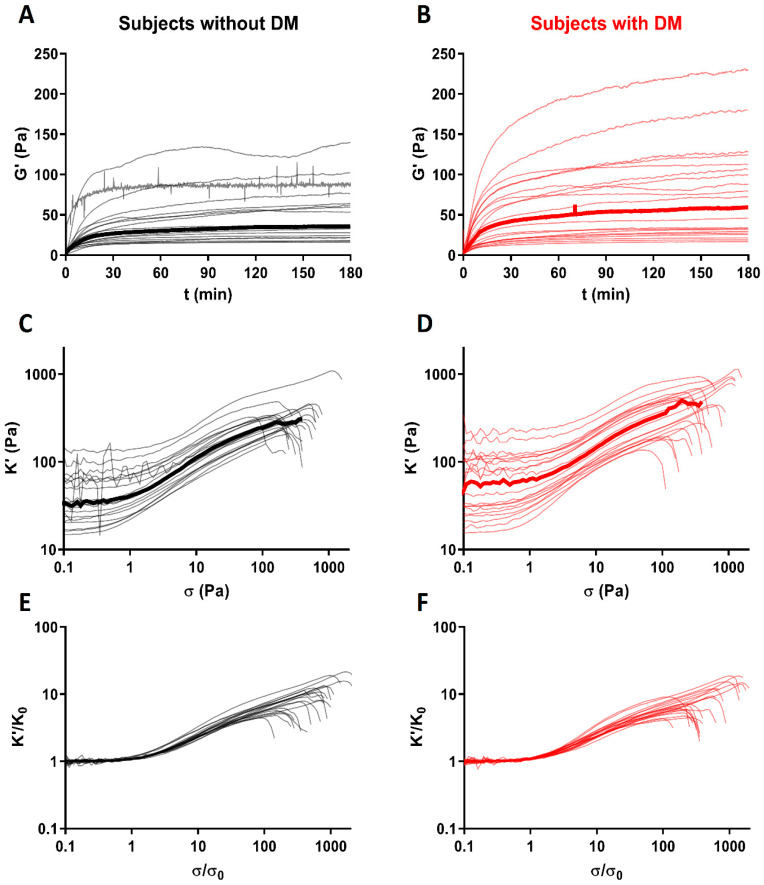
Rheology of clots prepared from plasma from subjects without or with diabetes with the addition of neutrophil extracellular traps (NETs). Thin lines represent individual measurements, thick line represents median. (**A**,**B**) The linear elastic modulus (G’) at low strain (0.5%) is shown as a function of time. (**C**,**D**) The differential modulus (K’) is shown as a function of applied shear stress (σ) to show the strain-stiffening behaviour of the plasma clots. (**E**,**F**) Nonlinear response of plasma clots, K’ is normalized by the linear modulus, K_0_, and shear stress is normalized by the onset stress where strain-stiffening sets in (σ_0_).

In the rheology experiments, neutrophils were activated with PMA instead of *S. aureus.* Using parallel confocal imaging, we checked the formation of NETs and confirmed that NETs were visible within 3 h after activation. The linear elastic moduli, G’, of plasma clots prepared from subjects with DM showed more variation and were slightly, but not significantly, higher compared to plasma clots from subjects without DM (Figure 3A,B and Table S3). The median G’ was 62.1 [29.7–188.9] Pa in plasma clots from subjects with DM, while a median value of 36.4 [23.0–80.0] Pa was found in plasma clots of subjects without DM. Next, the response of the clots to large deformations was measured by increasing the shear stress. Similar strain-stiffening behavior was observed for both groups (Figure 3C,D). Again, a wider spread was observed in onset stress of strain-stiffening (σ_0_), maximum stiffness before rupture (K_max_), stress at K_max_ (σ_max_), and stress at point of rupture ((σ_R_) in plasma clots from subjects with DM compared to subjects without DM (Table S3). The maximum stiffness before rupture (K_max_) was slightly, but not significantly (*p* = 0.11), higher in plasma clots from subjects with DM (520.4 [248.6–827.6]) compared to plasma clots from subjects without DM (320.4 [285.0–405.6]) (Table S3). After adjustment for fibrinogen levels, the small differences between subjects with and without DM completely disappeared (Table 3). Furthermore, after normalization for K_0_ and onset stress of strain-stiffening (σ_0_), curves from subjects with and without DM completely overlapped (Figure 3E,F), indicating plasma clots from subjects with and without DM had a similar response to increasing shear stress. 

### 2.4. Fibrinolysis in Plasma clots with NETs in Subjects with and without DM

**Figure 4:**
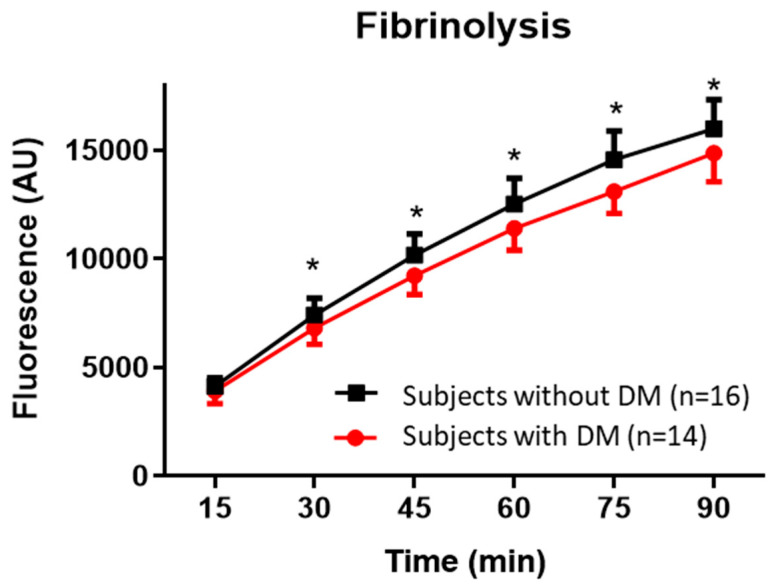
Fibrinolysis was significantly slower in clots prepared from plasma from subjects with diabetes mellitus (DM) with the addition of neutrophil extracellular traps (NETs) compared to clots prepared from plasma from subjects without DM. Clots were prepared in the presence of fibrinogen labeled with Alexa-Fluor 488. 100 ng/mL tPA was added after which small samples (20 µL) of the lysed product were taken every 15 min. Increased fluorescence corresponds to increased fibrinolysis and values of each time point are depicted as mean ± SD. Differences were analyzed by repeated measures ANOVA and comparisons at the different time points were adjusted for multiple comparisons (Bonferroni): * indicates *p*-value below 0.05.

While the degree of released fluorescence, representing the degree of fibrinolysis, at 15 min was comparable between plasma clots from subjects with and without DM, the degree of fibrinolysis after 90 min was significantly lower in subjects with DM, adjusted for fibrinogen (Β = −1236, 95% CI −2312; −159) (Table 3). This implies that the rate of fibrinolysis was slower in clots with added NETs prepared from plasma from subjects with DM compared to subjects without DM (Figure 4). Adding non-stimulated neutrophils or *S. aureus* to plasma clots had no effect on the rate of fibrinolysis compared to clots prepared from plasma only (Figure S1). 

Finally, the increased fibrinolysis after 90 min was correlated with decreased stiffness of clots (r_s_ = −0.68, *p* < 0.01), thinner fibrin fibers (r_s_ = −0.78, *p* < 0.01), smaller pores (r_s_ = −0.69, *p* < 0.05) and increased numbers of pores (r_s_ = 0.65, *p* < 0.05) (Table S1). 

### 2.5. Effect of Previous Arterial Thrombosis or Medication Use on Clot Characteristics

Because 61% of subjects had a history of arterial thrombosis, which has been reported to affect clot characteristics compared to healthy controls [6], we tested the influence of arterial thrombosis or medication use on clot characteristics in our study population. No differences in any of the clot parameters tested were observed between subjects in the current study with and without previous arterial thrombosis, neither in the total population nor in the subgroups of subjects with or without DM. We did find an increased fiber diameter in subjects using statins (193.14 ± 5.22 nm) compared to subjects without statin use (186.94 ± 4.64 nm; *p* = 0.03). Additional adjustment for previous arterial thrombosis, the use of statins or the use of aspirin in the regression analyses for the effect of diabetes on clot parameters did not change the results, suggesting our findings are independent of arterial thrombosis or medication use.

## 3. Discussion

Alterations in clot structure and properties may contribute to the increased risk of arterial thrombosis in subjects with DM. In this study, we investigated the structural and mechanical parameters of clots prepared from plasma from subjects with and without DM in a model of acute NETs formation. To our knowledge, this is the first study describing the effect of DM on the fibrin network after addition of NETs to resemble the acute phase of NETs formation present during disease in vivo. After adjustment for fibrinogen level, we observed significantly thicker fibrin fibers, a lower number of pores and decreased intersection density in clots prepared from plasma from subjects with DM compared to those without. In addition, after adjustment for fibrinogen level, fibrinolysis was significantly slower in clots prepared from plasma from subjects with DM. No differences were found in the mechanical parameters. 

### 3.1. Structural Clot Parameters Observed Using SEM Imaging

No differences in structural parameters between subjects with and without DM were found in clot prepared from plasma only. This is in line with previous studies in which no differences were found in fiber diameter between clots prepared from purified fibrinogen from subjects with and without DM [5,21]. Interestingly, in clots to which NETs were added, fibers in plasma clots from subjects with DM were observed to be thicker compared to fibers from subjects without DM, adjusted for fibrinogen concentration. Furthermore, a decreased number of pores and decreased intersection density was found in subjects with DM. The combination of thicker fibers, decreased number of pores and unchanged pore size suggest a more dense structure in clots from subjects with DM, as described in literature [4]. It should be taken into account that the quantification of pores and intersections was done in two-dimensional SEM images, which does not necessarily reflect the three-dimensional structure of the clots. The diameter size is most likely not affected by the small diameters of nonoverlapping NETs, since we do not observe a higher peak around the expected 10-20 nm for NETs fibers in the histograms of diameters from clots containing NETs compared to clots without NETs (Figure 2) [9,22]. The presence of the round bacteria with a diameter around 1000 nm did also not influence the results, since the DiameterJ plugin removes all intersections, including the bacteria, before determining the diameter of the fibers [23].

Significant positive correlations between fibrinogen, glucose or HbA1c levels and fiber diameter were found, corresponding to previous reports [5,20]. This shows that worsened glycaemic control, as shown by increased HbA1c levels, is associated with thicker fibers [5]. In addition, increased HbA1c levels were negatively associated with intersection density and the number of pores, suggesting worsened glycaemic control affects the structure of plasma clots [5]. 

### 3.2. Mechanical Parameters of Clots

We found a positive trend towards stiffer clots from plasma from subjects with DM, but no significant differences in mechanical properties between subjects with and without DM in clots with NETs formation. These slight differences completely disappeared after adjustment for the fibrinogen level. This finding corresponds to previous studies which found a similar stiffness of clots prepared from purified fibrinogen from subjects with DM or controls [21,24]. To our knowledge, there have been no other studies performed which compared the stiffness of plasma clots from subjects with and without DM. Interestingly, we do find a significant positive association between glucose levels and maximum stiffness of the clots before rupture, which suggests worsened glycaemic control in DM affects the nonlinear elastic properties of plasma clots. 

Furthermore, our results confirm previous studies by showing a positive correlation between the stiffness of clots and fibrinogen concentration [20,25,26]. In addition, higher fibrinogen levels were significantly associated with higher levels of onset stress for strain-stiffening, while the corresponding onset strain was negatively associated with fibrinogen levels. This suggests that clots made with higher fibrinogen concentrations start strain stiffening at a higher shear stress, but smaller deformations, corresponding to the increased stiffness. It is clinically relevant to have information about nonlinear mechanical properties of a clot, since these determine the response of the clot to the large mechanical stresses caused by blood flow. The clot can deform reversibly or irreversibly, rupture and embolize, affecting the occurrence and progression of thrombotic diseases and the response to treatment [27]. It is indeed shown that plasma clots from patients with a previous myocardial infarction are significantly stiffer compared to healthy controls [28]. Previously it was suggested that high fibrinogen levels translate into a higher risk of myocardial infarction via increased stiffness of formed clots [29]. 

### 3.3. Fibrinolysis

Finally, fibrinolysis was significantly slower in plasma clots from subjects with DM compared to plasma clots from subjects without DM, after adjustment for fibrinogen levels. It is known that fibrinolysis is impaired in subjects with DM, due to an altered clot structure and impaired fibrinolytic system [30]. Our finding shows that this decreased rate of fibrinolysis in clots prepared from subjects with DM is also found in a model of acute NETs formation and independent of fibrinogen level. In addition, we found a significant negative correlation between the rate of fibrinolysis and fibrinogen concentration and clot stiffness, confirming previous data [20].

### 3.4. Limitations

We acknowledge some possible limitations in the current study. Firstly, a small number of platelets was introduced in the plasma clots due to platelet contamination in the isolated neutrophils. However, this was only 2% of the normal concentration of platelets in the blood and previous studies show that this small number will not have a relevant effect on clot properties [31]. We chose to study plasma clots instead of clots formed from whole blood to focus our study on the effects on the fibrin network, without potential confounding by other cells. However, it must be taken into account that white blood cells or platelets from subjects with DM might behave differently compared to cells from subjects without DM, further affecting clot characteristics. As a consequence, before being able to fully translate this research to the in vivo situation, future studies on more complicated models will be required.

Secondly, we used different inducers of NETs formation between the different experiments. The bacterium *S.aureus*, used in SEM imaging and the fibrinolysis assay, induces NETs formation within min. *S. aureus* had our preference above the other powerful inducer of NETs formation, PMA, since PMA affects fibrin clot characteristics and is less physiological. Because it was not permitted to work with bacteria in the rheology experiments, we used PMA to activate neutrophils in these experiments. PMA is a non-physiological inducer of NETs, which is shown to induce NETs formation within a time range between 10 min and several h [32]. This difference in timing was taken into account by incubating the plasma clots with neutrophils and PMA for 3 h between the rheometer plates. Using parallel confocal imaging, we checked for the formation of NETs and confirmed that NETs were visible within 3 h after activation. It should be noted that the use of different NETs inducers might have affected the correlation of the mechanical parameters with the clot characteristics determined using SEM imaging or the fibrinolysis assay (Table S1). Furthermore, it is possible that because of the different timeframes of NETs formation for the different NETs inducers, there is a difference in the effect on clotting. Therefore, future studies should compare mechanical parameters of clots formed in the presence of NETs induced by either PMA or *S. aureus*. This was beyond the scope of this project. 

A final limitation is the study population. Subjects from the ATTAC study were used, of which 61% suffered from arterial thrombosis. However, we matched subjects with and without DM based on age, sex and the occurrence of arterial thrombosis. In addition, no effects of arterial thrombosis were found on any of the clot characteristics in this population. The groups in this study are also relatively small, therefore findings should be confirmed in bigger cohorts. Also the effects of DM in elderly subjects should be investigated, since the pathophysiology of DM might be somewhat different than in the relatively young subjects in the current study. Furthermore, in the group of subjects with DM, there were a few subjects with type 1 DM, while the majority of the subjects had type 2. However, exclusion of the subjects with type 1 DM (n = 2) and/or the subjects with unspecified type of diabetes (n = 6) did not alter our results. It must be taken into account that in the current study most subjects with DM use medication to control their disease, which might have affected properties of the fibrin network [21]. However, the HbA1c and glucose levels were significantly higher in subjects with DM compared to subjects without DM, suggesting this glycaemic control is not complete.

## 4. Materials and Methods

### 4.1. Plasma Samples

Plasma samples from 46 subjects included in the Genetic Risk Factors for Arterial Thrombosis at a Young Age: the role of TAFI and other Coagulation Factors (ATTAC) study were studied. In the ATTAC study, young patients (men ≤45 years and women ≤55 years old) with arterial thrombosis (myocardial infarction or ischemic stroke) and healthy controls were included in the Erasmus Medical Center Rotterdam in the Netherlands [33]. Blood was collected at least one month after the cardiovascular event into 3.2% trisodium citrate tubes (9:1 *vol*/*vol*) and centrifuged within 1 h at 2000 *g* and 20,000× *g* for 10 min at 4 °C. The obtained platelet-poor plasma was stored at −80 °C until use. Levels of fibrinogen, glucose and glycated hemoglobin (HbA1c) were determined using routine clinical chemical methods. Subjects were classified as having diabetes mellitus (DM) when a medical history of DM was reported or insulin or anti-diabetic medication was used. From the ATTAC study, 20 subjects with DM were randomly selected for the current study. 2 subjects had DM type 1, 12 subjects had DM type 2 and for 6 subjects the type was not clearly specified. Age- and sex-matched subjects without DM were used as controls (*n* = 26). In addition, subjects with and without DM were matched based on the occurrence of arterial thrombosis. Written informed consent was obtained from all participants of the ATTAC study and the study was approved by the Medical Ethics Committee of Erasmus MC (MEC 198/2001-23) (2001) and conducted according to the procedures of the Declaration of Helsinki. In the experiments with pooled control plasma, VisuCon-F Frozen Unassayed Normal Control Plasma (UFNCP0181, Affinity Biologicals, Ancaster, ON, Canada) was used.

### 4.2. Neutrophil Isolation

Neutrophils were freshly isolated from citrated blood from healthy volunteers using density gradient medium Lymphoprep (Stem Cell Technologies, Grenoble, France). Plasma was removed after centrifuging the blood at 2000× *g* for 10 min. The remaining cells were diluted 1:1 with PBS (phosphate buffered saline without Ca_2_^+^/Mg, 17-516F, Lonza, Walkersville, IL, USA) and loaded on top of 15 mL density gradient Lymphoprep. After centrifugation at 830× *g* for 15 min, the upper layer containing plasma, white blood cells and Lymphoprep was removed and the pellet containing red blood cells and granulocytes was incubated with 40 mL erythrolysis buffer (3.1M NH_4_Cl, 0.2M KHCO_3_, 0.02M EDTA, pH 7.4) for 10 min. This step was repeated to remove all erythrocytes. Following centrifugation at 690× *g* for 8 min, cells were washed with HEPES buffer (0.115M NaCl, 0.01M HEPES, pH 7.4) and centrifuged at 690× *g* for 8 min. Neutrophils were counted, centrifuged at 0.5× *g* for 2 min and resuspended in plasma from the subjects used in this study. The neutrophil isolate contained some platelets, resulting in about 6 × 10^6^ platelets/mL in the clots. 

### 4.3. Scanning Electron Microscopy

Citrated plasma was thawed for 10 min at 37 °C and centrifuged at 2000× *g* for 5 min to prevent debris in the clot. To induce NETs formation, neutrophils (2 × 10^6^/mL) in plasma were activated by adding *Staphylococcus aureus* Newman bacteria (2 × 10^9^/mL), which was incubated for 5 min at RT before clotting was induced [32]. Clotting was induced by adding calcium (CaCl_2_, final concentration 25 mM) and human thrombin (final concentration 0.5 U/mL, T7009, Sigma Aldrich, Zwijndrecht, The Netherlands) to plasma. The clots were left to form for 30 min at RT and washed three times with cacodylate buffer (0.05 M cacodylic acid and 0.2 M HCl in distilled H_2_O) and fixed in 2% glutaraldehyde (G7651, Sigma Aldrich) in 0.05M cacodylate buffer for 2 h. The fixed clots were subsequently dehydrated in 30, 50, 70, 80, 90, and 95% ethanol and finally three times in 100% ethanol. After dehydration, drying was performed by adding 50% and subsequently 100% hexamethyldisilazane (440191, Sigma Aldrich, Zwijndrecht, The Netherlands), which was left to evaporate overnight. Samples were sputter coated with a layer of 15 nm gold/palladium using the Leica EM ACE600 (Leica, Wetzlar, Germany) and imaged using an FEI Verios 460 scanning electron microscope (Thermo Fisher Scientific, Waltham, MA, USA) at 10 kV. Images were taken at 5–randomly selected areas at 10,000 times magnification and analyzed using ImageJ (1.52p, Wayne Rasband, National Institutes of Health, Bethesda, MD, USA). 

To prepare the images for analysis, a bandpass filter was used to blur the images by filtering out intensity variations above 20 pixels and small structures below 3 pixels (Figure S2). Subsequently, the DiameterJ plugin was used to determine structural plasma clot properties [23]. SEM images were first segmented into a binary representation with black pixels corresponding to the background and white pixels to fibrin fibers, using the M2 segmentation algorithm from the plugin [23]. The M2 segmentation algorithm was chosen in this analysis, since this algorithm best represented the SEM images, both separating individual fibers and enabling quantification of pore size and intersections. With other segmentation algorithms, similar differences between subjects with and without DM were found (data not shown). Subsequently, the following parameters were determined using the DiameterJ plugin: mean diameter of all fibrin fibers per image (measured at every pixel along the fibers), mean pore area (mean number of black pixels in discrete clusters enclosed by fibers), number of pores, percentage porosity (number of black pixels divided by the total number of pixels), and intersection density (number of fiber overlaps divided by the total area of the image). 

### 4.4. Rheology

To assess the mechanical properties of the clots, clots were prepared between plates of a rheometer. Neutrophils were freshly isolated from blood from healthy volunteers and resuspended in plasma to a concentration of 2 × 10^6^/mL. To activate the neutrophils, 250 ng/mL PMA (stock 100 µg/mL in DMSO) was added to the neutrophils in plasma right before the induction of clotting. In the rheology experiments, PMA was used as activator instead of *S. aureus* bacteria because the use of bacteria was not allowed at the rheometer facilities. To induce clotting, 17 mM CaCl_2_ and 1 U/mL thrombin (final concentrations) were added and 350 μL of the sample was immediately transferred to a stress-controlled rheometer (MCR501, Anton Paar, Graz, Austria) with a steel cone and plate geometry (40-mm diameter, 1° cone angle). Mineral oil was applied around the sample edge to prevent water evaporation and all measurements were performed at 37 °C. To increase attachment of the plasma clots to the rheometer plates, the plates were coated with 1 mg/mL human fibrinogen (0.024 mg/cm^2^) (FIBI, Enzyme Research Laboratories, South Bend, IN, USA) for 30 min at 37 °C. Polymerization was studied by applying small strain oscillations with an amplitude of 0.5% and a frequency of 0.5 Hz for 3 h, while recording the storage modulus (G’) and loss modulus (G’’), which give information about the elastic and viscous properties of the clot, respectively and are also described as stiffness of the clot. The steady-state values of the shear moduli were determined by fitting an exponential function to the time-dependencies. The nonlinear rheology was studied using a stress ramp protocol, in which the shear stress was gradually increased from 0.01 Pa to 10,000 Pa with 20 points per decade (10 s per point) until the sample ruptured, as evidenced by a sudden drop in the elastic modulus. The differential modulus (K’) was computed by differentiating the stress/strain curve. Using a standardized script written in Python, the onset stress and strain where the linear regime ends and strain-stiffening begins (σ_0_ and γ_0_) (value where R^2^ of linear fit is below 0.8), the maximum value that K’ reaches before network rupture (K_max_), the stress and strain values at K_max_ (σ_max_ and γ_max_), and the stress and strain values at the point of rupture (σ_R_ and γ_R_) were determined (Figure S3).

### 4.5. Fibrinolysis Assay

Neutrophils were freshly isolated from blood from healthy volunteers and added to 200 μL subject plasma (5 × 10^6^ neutrophils/mL) together with 5 × 10^8^ *S. aureus* bacteria/mL. Finally, 0.1 mg/mL AF488-labeled fibrinogen (F13191, Thermo Fisher Scientific) was added and clotting was induced by 17 mM CaCl_2_ and 1 U/mL thrombin (final concentrations). Clots were formed in Eppendorf tubes coated with TBS/1% Tween 20 (50 mM Tris, 100 mM NaCl and 1% Tween 20 in distilled H_2_O, pH 7.4) to prevent adhesion to the wall. Clots were incubated at 37 °C for 1.5 h, after which 1 mL of 0.1 μg/mL tissue-type plasminogen activator (tPA) in assay buffer (25 mM HEPES, 137 mM NaCl, 3.5 mM KCL, 1% BSA, pH 7.4) was added to induce clot lysis. Small samples (20 μL) of the lysed product were taken every 15 min while samples were on a shaker at 750 rpm and 37 °C. The samples were 12-fold diluted in TBS/0.01% Tween 20 and fluorescence was measured at 37 °C using a microplate reader (Victor^TM^ multilabel counter, Perkin Elmer, Turku, Finland). Because fibrinolysis results in the release of the AF488 label from the clot into the liquid, the amount of fluorescence in every sample is a measure for the amount of degraded fibrin.

### 4.6. Statistical Analysis

Differences between subjects with DM and without DM were tested using an independent students’ *t*-test (normally distributed data), Mann-Whitney U test (not-normally distributed data) or Chi-square test (categorical data). Spearman’s correlation was used to test correlations between the parameters and levels of fibrinogen, glucose or HbA1c. Differences between subjects with and without DM in the fibrinolysis assay were tested by repeated measures ANOVA with Bonferroni correction. In addition, associations between clot properties and DM were tested using linear regression with (log-transformed) clot properties as dependent variable and DM as independent variable, adjusted for fibrinogen levels. Statistical analyses were performed using IBM SPSS Statistics v25 (IBM, Armonk, NY, USA) and a *p*-value below 0.05 was considered as statistically significant. 

## 5. Conclusions

In conclusion, in a model resembling an acute phase of disease with NETs formation, plasma clots from subjects with DM had thicker fibrin fibers and a decreased number of pores and branch points. Fibrinolysis was significantly reduced in plasma clots from subjects with DM compared to subjects without DM, while no differences were found in the mechanical properties of the plasma clots. These results implicate that DM has prothrombotic effects in acute situations with NETs formation, further elucidating the mechanism behind the increased risk of arterial thrombosis in DM. 

## Figures and Tables

**Table 1:** Characteristics of subjects.

Characteristic	Total (*n* = 46)	DM (*n* = 20)	Without DM (*n* = 26)	*p*-Value
Age (years)	46.4 ± 5.7	46.1 ± 6.2	46.6 ± 5.4	0.77
Gender (male)	16 (35%)	7 (35%)	9 (35%)	0.98
Body mass index	28.4 [24.7–32.4]	29.9 [27.0–36.5]	27.0 [24.2–29.5]	0.03 *
Smoking	17 (37%)	6 (30%)	11 (42%)	0.39
Cardiovascular event	28 (61%)	14 (70%)	14 (54%)	0.27
Subtype event				0.75
AMI	12 (43%)	6 (43%)	6 (43%)	
TIA	5 (18%)	3 (21%)	2 (14%)	
IS	10 (36%)	5 (36%)	5 (36%)	
UAP	1 (4%)	0 (0%)	1 (7%)	
Use of statins	31 (67%)	17 (85%)	14 (54%)	0.03 *
Use of aspirin	27 (59%)	14 (70%)	13 (50%)	0.17
Fibrinogen (g/L)	3.5 ± 0.7	3.8 ± 0.7	3.3 ± 0.6	0.01 *
HbA1c (%)	5.8 [5.4–7.4]	7.4 [6.8–8.6]	5.4 [5.3–5.7]	<0.01 *
Glucose (mmol/L)	6.0 [5.0–9.3]	8.5 [6.8–11.5]	5.0 [5.0–5.0]	<0.01 *

Values are given as *n* (%) or as mean ± SD for normally distributed variables or median [25th–75th percentile] for not normally distributed variables. *p*-values are given for the difference between subjects with and without diabetes mellitus (DM) as tested by the students’ *t*-test, Mann-Whitney U test or Chi-square test. * indicates *p*-value below 0.05. AMI, acute myocardial infarction; DM, diabetes mellitus; HbA1c, glycated hemoglobin; IS, ischemic stroke; TIA, transient ischemic attack; UAP, unstable angina pectoris.

**Table 2:** Correlation between characteristics of clots prepared in the presence of NETs and the levels of fibrinogen, glucose and HbA1C in the total study population.

Clot Characteristic	Fibrinogen		Glucose		HbA1c	
	r_s_	*p*	r_s_	*p*	r_s_	*p*
Glucose	0.18	0.39				
HbA1c	0.21	0.26	0.79	<0.01 *		
Fiber diameter	0.60	0.01 *	0.74	0.04 *	0.83	0.01 *
Porosity	0.29	0.28	0.42	0.31	0.45	0.26
Pore area	0.37	0.16	0.61	0.11	0.50	0.21
Number of pores	−0.49	0.06	−0.66	0.08	−0.71	0.05 *
Intersection density	−0.47	0.07	−0.61	0.11	−0.74	0.04 *
G’	0.55	<0.01 *	0.26	0.20	0.18	0.35
G’’	0.63	<0.01 *	0.26	0.20	0.23	0.22
σ_0_	0.56	<0.01 *	0.35	0.09	0.22	0.26
γ_0_	−0.35	0.03 *	−0.07	0.75	−0.01	0.95
K_max_	0.56	<0.01 *	0.41	0.04 *	0.32	0.09
σ_max_	0.19	0.26	0.29	0.15	0.29	0.12
γ_max_	−0.25	0.12	−0.16	0.43	0.03	0.89
σ_R_	0.18	0.28	0.27	0.19	0.30	0.11
γ_R_	−0.40	0.01 *	−0.14	0.50	−0.04	0.85
Fibrinolysis	−0.52	<0.01 *	−0.51	0.09	−0.24	0.32

* indicates *p*-value below 0.05. G’, storage modulus; G’’, loss modulus; HbA1c, glycated hemoglobin; K_max_, maximum differential storage modulus; NETs, neutrophil extracellular traps; r_s_; Spearman’s correlation coefficient; σ_0_, onset stress of strain-stiffening; σ_max_, stress at K_max_; σ_R_, stress at point of rupture; γ_0_, onset strain of strain-stiffening; γ_max_, strain at K_max_; γ_R_, strain at point of rupture.

**Table 3:** Association between diabetes mellitus (DM) and the mechanical and structural parameters of plasma clots in the presence of neutrophil extracellular traps (NETs).

Clot Characteristic	Unadjusted		Adjusted for Fibrinogen Level	
	B [95% CI]	*p*	B [95% CI]	*p*
Fiber diameter	8.46 [4.29;12.62]	<0.01 *	6.70 [1.53;11.87]	0.02 *
Porosity	0.02 [−0.01;0.04]	0.15	0.02 [−0.02;0.05]	0.31
Pore area	0.06 [0.02;0.10]	0.01 *	0.05 [−0.00;0.10]	0.07
Number of pores	−290.4 [−447.6;−133.2]	<0.01 *	−268.0 [−472.6;−63.4]	0.01 *
Intersection density	−0.49 [−0.75;−0.22]	<0.01 *	−0.43 [−0.78;−0.09]	0.02 *
G’ ^†^	0.38 [−0.10;0.85]	0.11	0.05 [0.38;0.47]	0.83
G’’ ^†^	0.28 [−0.07;0.63]	0.11	0.02 [−0.29;0.32]	0.92
σ_0_^†^	0.37 [−0.01;0.74]	0.05	0.14 [−0.22;0.50]	0.45
γ_0_^†^	−0.11 [−0.39;0.18]	0.47	0.04 [−0.25;0.33]	0.77
K_max_^†^	0.32 [−0.03;0.67]	0.07	0.09 [−0.24;0.42]	0.57
σ_max_^†^	0.17 [−0.38;0.71]	0.54	0.05 [−0.54;0.63]	0.87
γ_max_^†^	−0.17 [−0.55;0.21]	0.37	−0.05 [−0.45;0.36]	0.82
σ_R_^†^	0.15 [−0.29;0.59]	0.50	0.06 [−0.41;0.53]	0.80
γ_R_^†^	−0.23 [−0.56;0.11]	0.18	−0.07 [−0.41;0.27]	0.69
Fibrinolysis	−1551 [−2513;−589]	<0.01 *	−1236 [−2312;−159]	0.03 *

Linear regression with (log-transformed) parameters as dependent variable and DM as independent variable. * indicates *p*-value below 0.05. ^†^ All mechanical parameters were log-transformed. G’, linear storage modulus; G’’, linear loss modulus; K_max_, maximum differential storage modulus; σ_0_, onset stress of strain-stiffening; σ_max_, stress at K_max_; σ_R_, stress at point of rupture; γ_0_, onset strain of strain-stiffening; γ_max_, strain at K_max_; γ_R_, strain at point of rupture.

## Supplementary Materials

Supplementary materials can be found at https://www.mdpi.com/1422-0067/21/19/7107/s1.

## Abbreviations

σ_0_	Onset stress of strain-stiffening
σ_max_	Stress at maximum differential storage modulus
σ_R_	Stress at point of rupture
γ_0_	Onset strain of strain-stiffening
γ_max_	Strain at maximum differential storage modulus
γ_R_	Strain at point of rupture
ATTAC	Genetic risk factors for Arterial Thrombosis at a young age: the role of TAFI and other Coagulation factors
B	Beta coefficient
BMI	Body mass index
CaCl_2_	Calcium chloride
DM	Diabetes mellitus
G’	Linear storage modulus
G’’	Linear loss modulus
HbA1c	Glycated hemoglobin
K’	Differential modulus
K_max_	Maximum differential storage modulus
NETs	Neutrophil extracellular traps
PMA	Phorbol myristate acetate
r_s_	Spearman’s correlation coefficient
tPA	Tissue-type plasminogen activator
SEM	Scanning electron microscopy
