# Supplementary material for: Effects of Diabetes Mellitus on Fibrin Clot Structure and Mechanics in a Model of Acute Neutrophil Extracellular Traps (NETs) Formation
Source: Int J Mol Sci. 2020 Sep 26;21(19):7107. doi: 10.3390/ijms21197107 (PMC7582521; doi:10.3390/ijms21197107)
Supplement: Supplementary file 1 [file ijms-21-07107-s001.pdf]

## Supplemental data

**Supplemental Table S1.** Correlation between clot characteristics in the total study population in clots with added NETs

| | Fiber diameter | Porosity | Mean pore area | Number of pores | Intersection density | Stiffness (G') | Fibrinolysis at 90 minutes |
|---|---|---|---|---|---|---|---|
| **Fiber diameter** | | 0.22 | 0.50* | -0.69** | -0.74** | 0.39 | -0.78** |
| **Porosity** | 0.22 | | 0.92** | -0.82** | -0.78** | 0.27 | -0.42 |
| **Mean pore area** | 0.50* | 0.92** | | -0.95** | -0.92** | 0.41 | -0.69* |
| **Number of pores** | -0.69** | -0.82** | -0.95** | | 0.98** | -0.37 | 0.65* |
| **Intersection density** | -0.74** | -0.78** | -0.92** | 0.98** | | -0.33 | 0.62 |
| **Stiffness** | 0.39 | 0.27 | 0.41 | -0.37 | -0.33 | | -0.68** |
| **Fibrinolysis at 90 min** | -0.78** | -0.42 | -0.69* | 0.65* | 0.62 | -0.68** | |

Spearman's correlation coefficients are reported. * p<0.05, ** p<0.01

**Supplemental Table S2.** Structural characteristics of plasma clots from subjects with and without DM

| **Plasma clots with added NETs** | **Subjects without DM (n=8)** | **Subjects with DM (n=8)** | **p-value** |
|---|---|---|---|
| Fiber diameter (nm) | 186.6 ± 3.9 | 195.0 ± 3.9 | **<0.01*** |
| Porosity (%) | 0.45 ± 0.03 | 0.47 ± 0.02 | 0.15 |
| Mean pore area (μm$^2$) | 0.26 ± 0.04 | 0.31 ± 0.04 | **0.01*** |
| Number of pores | 1907.1 ± 169.0 | 1616.7 ± 120.0 | **<0.01*** |
| Intersection density (intersections/μm$^2$) | 3.44 ± 0.29 | 2.95 ± 0.21 | **<0.01*** |
| **Plasma clots without NETs** | **Subjects without DM (n=10)** | **Subjects with DM (n=9)** | **p-value** |
| Fiber diameter (nm)† | 207.5 [199.6-222.4] | 209.1 [203.3-214.0] | 0.66 |
| Porosity (%)† | 0.44 [0.42-0.45] | 0.44 [0.41-0.45] | 0.78 |
| Mean pore area (μm$^2$)† | 0.28 [0.23-0.34] | 0.26 [0.23-0.31] | 0.72 |
| Number of pores | 1710.2 ± 308.1 | 1671.3 ± 435.4 | 0.82 |
| Intersection density (intersections/μm$^2$) | 3.11 ± 0.55 | 3.05 ± 0.72 | 0.84 |

*p-value below 0.05, †variable was not normally distributed, median [IQR], Mann-Whitney U test

**Supplemental Table S3**. Differences in mechanical parameters of fibrin clots prepared from plasma from subjects with and without DM with added NETs

| | Without DM (n=20) | DM (n=20) | p-value |
|---|---|---|---|
| **G' (Pa)** | 36.4 [23.0-80.0] | 62.1 [29.7-188.9] | 0.23 |
| **G'' (Pa)** | 1.4 [1.0-2.0] | 1.6 [1.2-3.1] | 0.19 |
| **$\sigma_0$ (Pa)** | 0.71 [0.45-0.79] | 0.84 [0.45-1.54] | 0.13 |
| **$\gamma_0$ (%)** | 1.72 [1.30-2.22] | 1.91 [1.29-2.15] | 1.00 |
| **$K_{max}$ (Pa)** | 320.4 [285.0-405.6] | 520.4 [248.6-827.6] | 0.11 |
| **$\sigma_{max}$ (Pa)** | 251.2 [145.6-446.7] | 303.0 [168.8-671.5] | 0.50 |
| **$\gamma_{max}$ (%)** | 120.4 [59.6-183.7] | 78.8 [54.7-188.6] | 0.63 |
| **$\sigma_R$ (Pa)** | 446.7 [281.8-708.0] | 531.8 [316.2-972.8] | 0.43 |
| **$\gamma_R$ (%)** | 215.0 [139.3-293.7] | 173.6 [116.4-238.3] | 0.32 |

Median [$25^{th}$ – $75^{th}$ percentile], differences were tested with the Mann-Whitney U test. DM, diabetes mellitus; G', storage modulus; G'', loss modulus; $K_{max}$, maximum differential storage modulus; NETs, neutrophil extracellular traps; $\sigma_0$, onset stress of strain-stiffening; $\sigma_{max}$, stress at $K_{max}$; $\sigma_R$, stress at point of rupture; $\gamma_0$, onset strain of strain-stiffening; $\gamma_{max}$, strain at $K_{max}$; $\gamma_R$, strain at point of rupture.

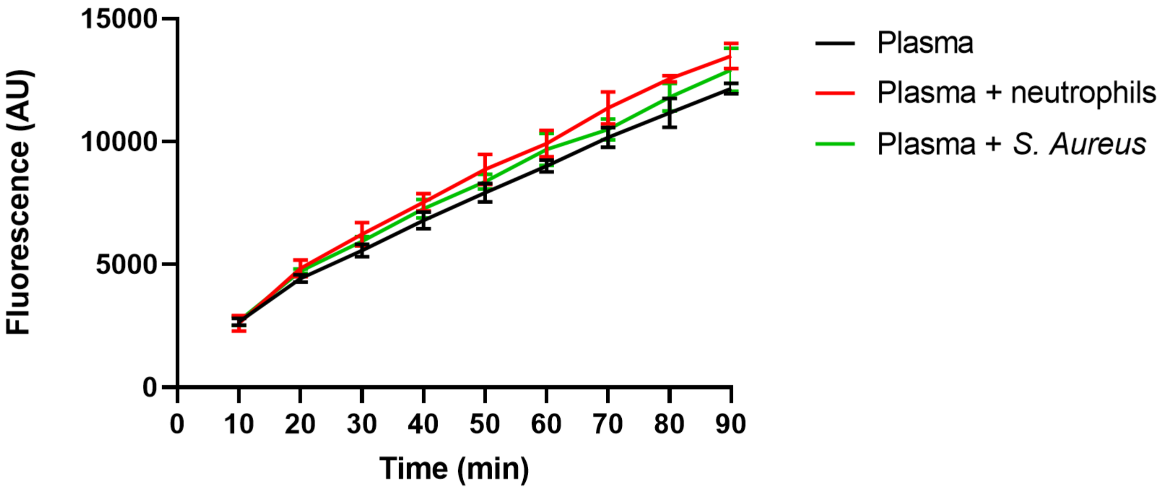


**Supplemental Figure S1**. The rate of fluorescence was comparable between clots prepared from pooled control plasma only, plasma with non-stimulated neutrophils or *S. Aureus*. Clots were prepared in the presence of fibrinogen labeled with Alexa-Fluor 488. 100 ng/ml tPA was added after which small samples (20 µl) of the lysed product were taken every 10 minutes. Increased fluorescence in these samples corresponds to increased fibrinolysis. Values of each time point are depicted as mean ± SD of n=3 clots. Differences were analyzed by repeated measures ANOVA and comparisons at the different time points were adjusted for multiple comparisons (Bonferroni).

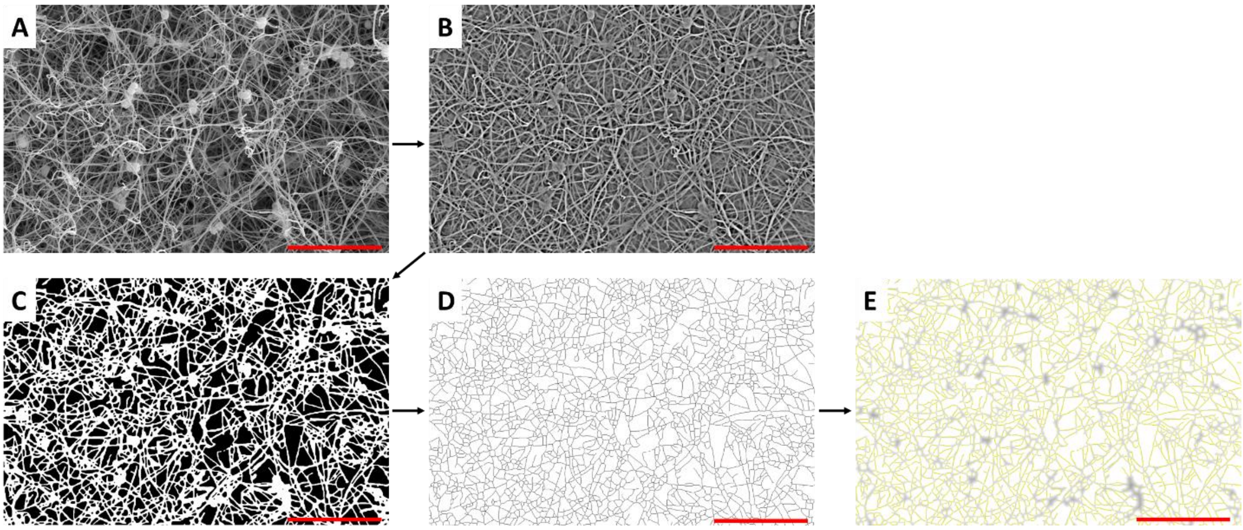


**Supplemental Figure S2.** Processing of scanning electron microscope images of fibrin clots using DiameterJ.

Scanning electron microscope images, scale bars 10 µm. The images (A) were blurred using a bandpass filter by filtering out intensity variations above 20 pixels and small structures below 3 pixels (B). Subsequently, the images were binarized using the M2 segmentation method of the DiameterJ plugin (C). These images were used to quantify the porosity, pore size and number of pores. From the binarized images, the centerline of the fibers was determined (D), which was used to quantify the amount of intersections. Finally, the intersections were removed to be able to quantify the diameters of the fibers (E). The removed intersections are depicted as gray spots, while the fibers of which the diameters were determined are depicted in yellow.

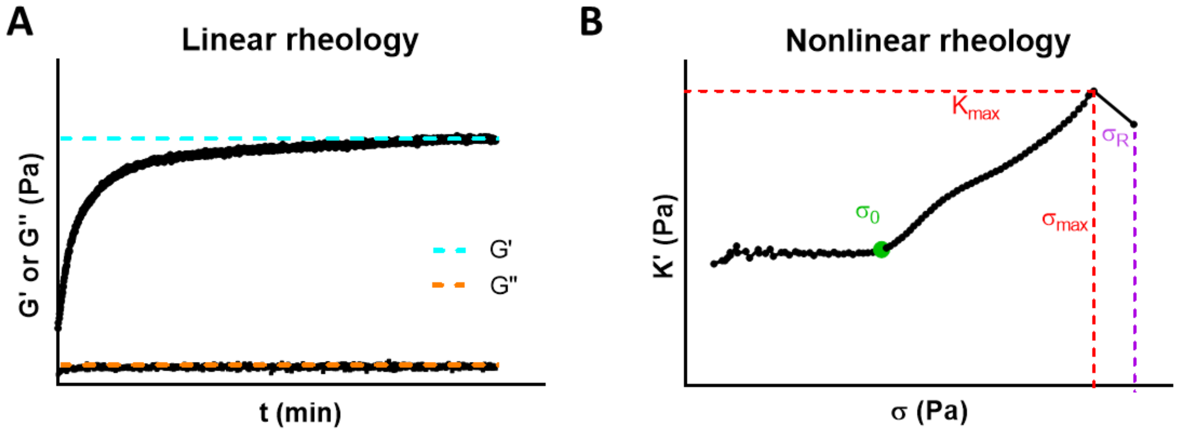


**Supplemental Figure S3**. Analysis of mechanical parameters of fibrin clots.

(A) The storage modulus (G') and loss modulus (G'') after polymerization were determined by fitting an exponential function to the time-dependencies recorded during clot formation between the plates of the rheometer. The dashed lines show the steady state values of G' and G''. (B) Using a standardized script written in Python, the onset stress ($\sigma_0$; green dot) and strain of strain-stiffening ($\gamma_0$) was determined from stress-stiffening curves by taking the value where $R^2$ of the linear fit is below 0.8. In addition, the maximum value of K' ($K_{max}$) and the corresponding stress ($\sigma_{max}$; dashed red lines) and strain ($\gamma_{max}$) values in addition to the stress ($\sigma_R$; dashed purple line) and strain ($\gamma_R$) values at the point of rupture were determined.
